# Transmission Dynamics of the Four Dengue Serotypes in Southern Vietnam and the Potential Impact of Vaccination

**DOI:** 10.1371/journal.pone.0051244

**Published:** 2012-12-10

**Authors:** Laurent Coudeville, Geoff P. Garnett

**Affiliations:** 1 Dengue Company, Sanofi Pasteur, Lyon, France; 2 Department of Infectious Disease Epidemiology, School of Public Health, Imperial College, London, United Kingdom; Duke-National University of Singapore Graduate Medical School, Singapore

## Abstract

**Background:**

With approximately 2.5 billion people at risk, dengue is a major international public health concern. Dengue vaccines currently in development should help reduce the burden associated with this disease but the most efficient way of using future dengue vaccines remains to be defined. Mathematical models of transmission can provide insight into the expected impact of different vaccination strategies at a population level and contribute to this definition.

**Methods and Findings:**

We developed and analyzed an age-structured, host-vector and serotype-specific compartmental model, including seasonality. We first used this transmission model to identify the immunological interactions between serotypes that affect the risks and consequences of secondary infections (cross-protection, increased susceptibility, increased severity, and increased infectiousness) and reproduce the observed epidemiology of dengue. For populating this model, we used routine surveillance data from Southern Vietnam and the results of a prospective cohort study conducted in the same area. The model provided a good fit to the observed data for age, severity of cases, serotype distribution, and dynamics over time, using two scenarios of immunological interaction : short term cross-protection alone (6–17 months) or a combination of short term cross-protection with cross-enhancement (increased susceptibility, severity and infectiousness in the case of secondary infections). Finally, we explored the potential impact of vaccination for these two scenarios. Both highlighted that vaccination can substantially decrease dengue burden by reducing the magnitude and frequency of outbreaks.

**Conclusion:**

Our model suggests that seasonality and short term cross-protection are key factors for explaining dengue dynamics in Southern Vietnam. Vaccination was predicted to significantly reduce the disease burden, even in the situation where immunological cross-enhancement affects the risks and consequences of secondary infections.

## Introduction

Dengue disease is caused by four distinct serotypes of dengue virus. The viruses, members of the *Flavivirus* genus, circulate in tropical and subtropical regions of the world and are transmitted to humans by mosquitoes, primarily *Aedes aegypti* and *Aedes albopictus* [1], [2]. Given the geographic distribution of these vectors, approximately 2.5 billion people are at risk of infection [3]. During the 1990’s, up to 100 million people per year were infected, with up to half a million developing dengue hemorrhagic fever (DHF) [1]. Although efforts, including mosquito control and improved clinical management, contributed to reduce dengue burden, this disease remains a major public health concern in Asia and in the Americas [4]. No licensed vaccine is currently available to prevent this disease but several candidates are being developed [5] including one that has entered phase 3 clinical development [6]. Mathematical models of dengue transmission can, in this context, inform on the potential impact of vaccination [7].

The transmission dynamics of dengue has generated a lot of scientific interest, partly because primary exposure to one serotype is thought to alter the course of secondary, hetero-serotypic infections. The first mathematical model of dengue transmission, designed by Fischer and Halstead [8], and most analyses performed since, have focused on the effect of immunological interactions between heterotypic infections on disease dynamics. The types of interaction considered include cross-protection and cross-enhancement. Cross-protection, which decreases the risk that an individual exposed to one dengue serotype becomes infected by another serotype [9]–[13], is short-lived and provided by high-titer, heterotypic neutralizing antibodies that bind to the viral envelope (E) protein [14]. Cross-enhancement increases the susceptibility of developing secondary heterologous infections, as well as the severity of these secondary infections and the infectiousness of the host. It is hypothesized that this is caused by antibody-dependent enhancement (ADE), where non-neutralizing, heterotypic antibodies or sub-neutralizing levels of homotypic antibodies form virion-antibody complexes that facilitate cell entry [9], [10], [12], [13], [15]–[22]. Cross-protection and cross-enhancement are not mutually exclusive and some authors have considered models in which individuals benefit from a short period of cross-protection, before being exposed to cross-enhancement [9], [12], [13].

There are other differences between published models of dengue transmission. With the notable exception of the agent-based model developed by Focks et al. [11], most are compartmental models based on a set of differential equations [8]–[10], [12]–[13], [15]–[19], [21]–[23]. Only one model [11] includes a detailed representation of the vector population with their different stages of development (egg, larva, and pupa, adult). Most but not all represent the four dengue serotypes [8], [9], [15], [18]–[23] and some assume that an individual can be infected no more than twice [16], [21]. Only a few models consider age [16], [22], [23], despite the observed increase in the age of dengue cases in Thailand for example [24]. Dengue vaccination has been analyzed in several economic models [25]–[27] but to date no model has explored in detail the impact of vaccination on dengue transmission.

With the aim of understanding the potential impact of a dengue vaccine on a population, we explored the characteristics of a mathematical model of the transmission of the four dengue serotypes and its ability to reproduce the epidemiology observed in Southern Vietnam.

## Methods

A mathematical model representing the transmission dynamics of the four dengue serotypes in the human and mosquito populations of Southern Vietnam was developed and analyzed. Here we focus on the main features of the model and the epidemiological data used for its validation. Text S1 provides a more detailed presentation of the model.

### Model Design

**Figure 1:**
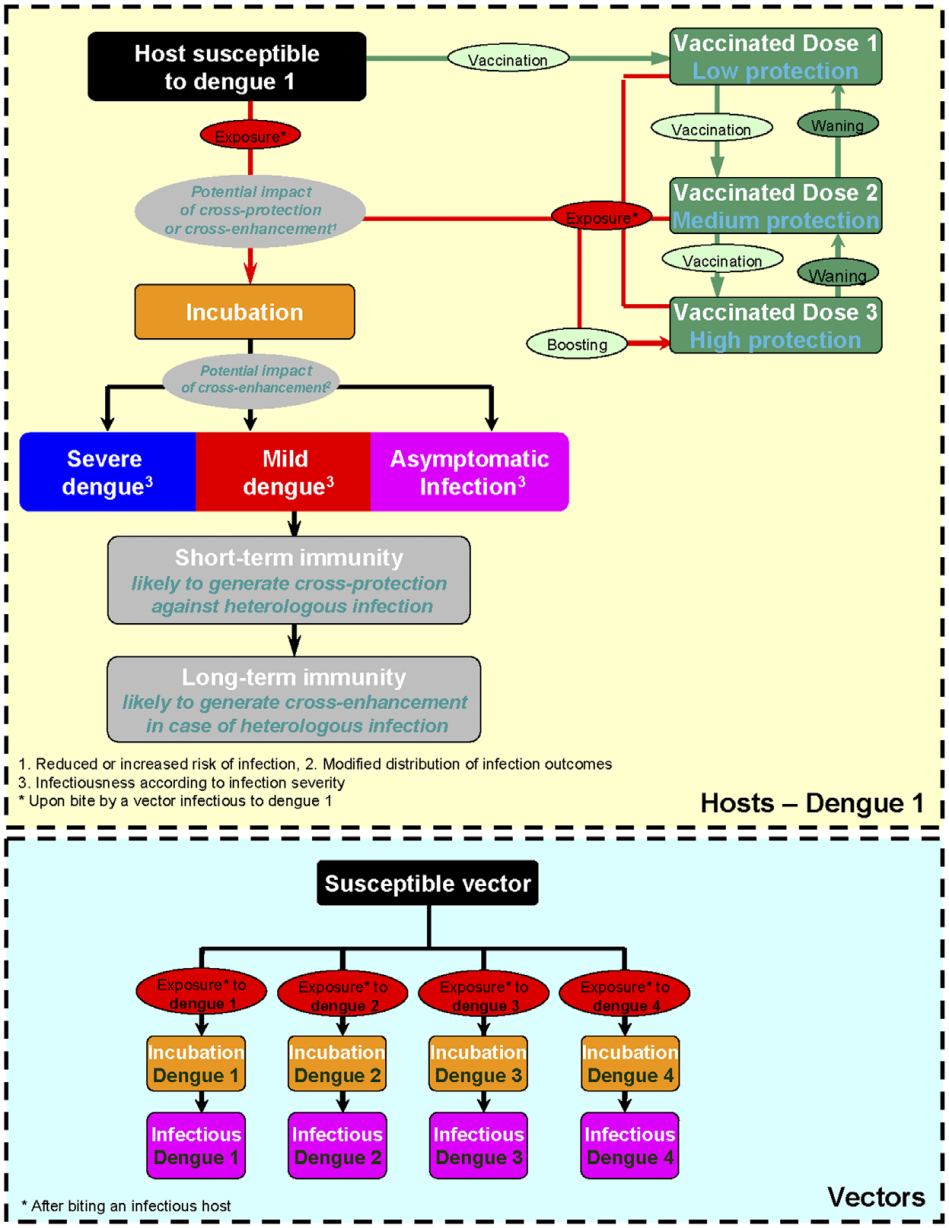
Flow diagram of the infection and vaccination process. Rounded rectangles correspond to compartments and ellipses to factors influencing the transition from one compartment to another. For clarity, the representation in human hosts is limited to serotype 1 but each individual is characterized by their status for each of the four serotypes.

Building upon a host-vector model of dengue that focused on the interactions between two serotypes [9], we constructed a deterministic, compartmental model capturing features known to affect the transmission dynamics of dengue: host-vector interactions; immunological interactions between the 4 dengue serotypes; population age structure; age-specific levels of transmission; seasonality of the disease, and the growth of the human and vector populations (Figure 1).

We assumed that all infants are born susceptible to the four dengue serotypes, and that maternal antibodies play no role. While maternal antibodies affect the risk of contracting dengue during infancy, the impact of this simplifying assumption on the overall disease dynamics is likely to be minimal [28]. Without vaccination, individuals become infected after being bitten by an infectious mosquito. After an incubation period that follows an exponential distribution and lasts 5 days on average, three possible outcomes of infection were considered: asymptomatic infection, mild disease, or severe disease.

Each infection is serotype-specific and provides lifelong, serotype-specific immunity, i.e., each individual can develop up to 4 dengue infections during their lifetime. The level of infectiousness of human hosts to biting vectors is likely to vary between severe, mild and asymptomatic infections as it depends upon viral load.

Consistent with the work reported by Bartley et al. [9], our model’s representation of the vector population is limited to adult, female mosquitoes. Seasonality was accounted for by varying the monthly growth rate of the vector population around an annual average. In most scenarios, we considered that this annual average was identical to that of the human population. Mosquitoes are initially susceptible to dengue and have a probability of becoming infected if they bite an infectious human. After a period of at least 8 days (10 on average), they become infectious for their remaining life. We assumed that a mosquito cannot be co-infected by more than one serotype.

We designed the model to be flexible to consider different types of immunological interactions between serotypes: temporary or permanent cross-protection, temporary or permanent cross-enhancement, or a combination of cross-protection and cross-enhancement.

Cross-protection was assumed to start immediately after the infectious period and prevents individuals from becoming infected by another serotype for a period ranging from 6 months to lifelong. In some scenarios, cross-protection was assumed to prevent only symptomatic disease but not asymptomatic infection.

Cross-enhancement was assumed to start immediately after the cross-protection period, or immediately after the infectious period when no cross-protection is considered. Three forms of cross-enhancement can be considered: increased susceptibility to infection by another serotype; increased disease severity of secondary infections (i.e., a higher proportion of severe infections only, or of both mild and severe infections), and increased infectiousness (through differences in infectiousness according to disease severity). We tested several scenarios for each form, corresponding to different levels of cross-enhancement in order to identify those likely to provide a good fit to observed data (see results and Text S2).

The consideration of the four dengue serotypes and a large variety of scenarios of cross-interactions led to a large number of compartments per age group (10,000 for hosts, 9 for vectors). However, since we excluded the possibility of co-infection by several serotypes, the vast majority of compartments therefore remained empty. For instance for all scenarios considering temporary cross-protection only 176 states in each age group contribute to dengue dynamics in the absence of vaccination and 1691 when vaccination is implemented. This simplified model excludes highly unlikely situations, such as the simultaneous co-infection by the 4 dengue serotypes.

Human demography was modeled according the methods adopted by Hethcote [29], [30] in his age-structured pertussis model in which individuals continuously flow between age groups. Both the host and vector populations were allowed to grow exponentially. In most scenarios we considered that the vector population grew at the same rate as the human population. The underlying assumption was that the size of the human population directly affects the size of the vector population as in predator-prey models [31]. We also considered that the age structure of the human population remains stable. This representation enabled us to derive an analytical solution for the steady state situation.

Like Bartley et al. [9], seasonality was modeled via monthly variations in the vector birth and biting rates. Although these two factors are known to play a role (see e.g. [32] for recent Thai data), other factors are also likely to impact dengue seasonality (e.g. variation of vector life expectancy, rate of development of a viral infection). Vector birth and biting rates are in fact used in the model as proxy variables to capture the seasonal variations in the observed size of the vector population and the incidence of dengue.

To parameterize and then validate the model, data describing different epidemiological variables from a single country or area are required. We chose to focus on Southern Vietnam, as for this area a comprehensive series of case reports is available, together with the results of a recent cohort study of disease and infection in children.

### Dengue Epidemiology in Southern Vietnam

Dengue is endemic throughout Vietnam, but transmission is highest in the south where very large epidemics occur regularly [33]. Dengue is a major public health concern in the area and substantial efforts are devoted to surveillance coordinated by the Pasteur Institute in Ho Chi Minh City.

**Figure 2:**
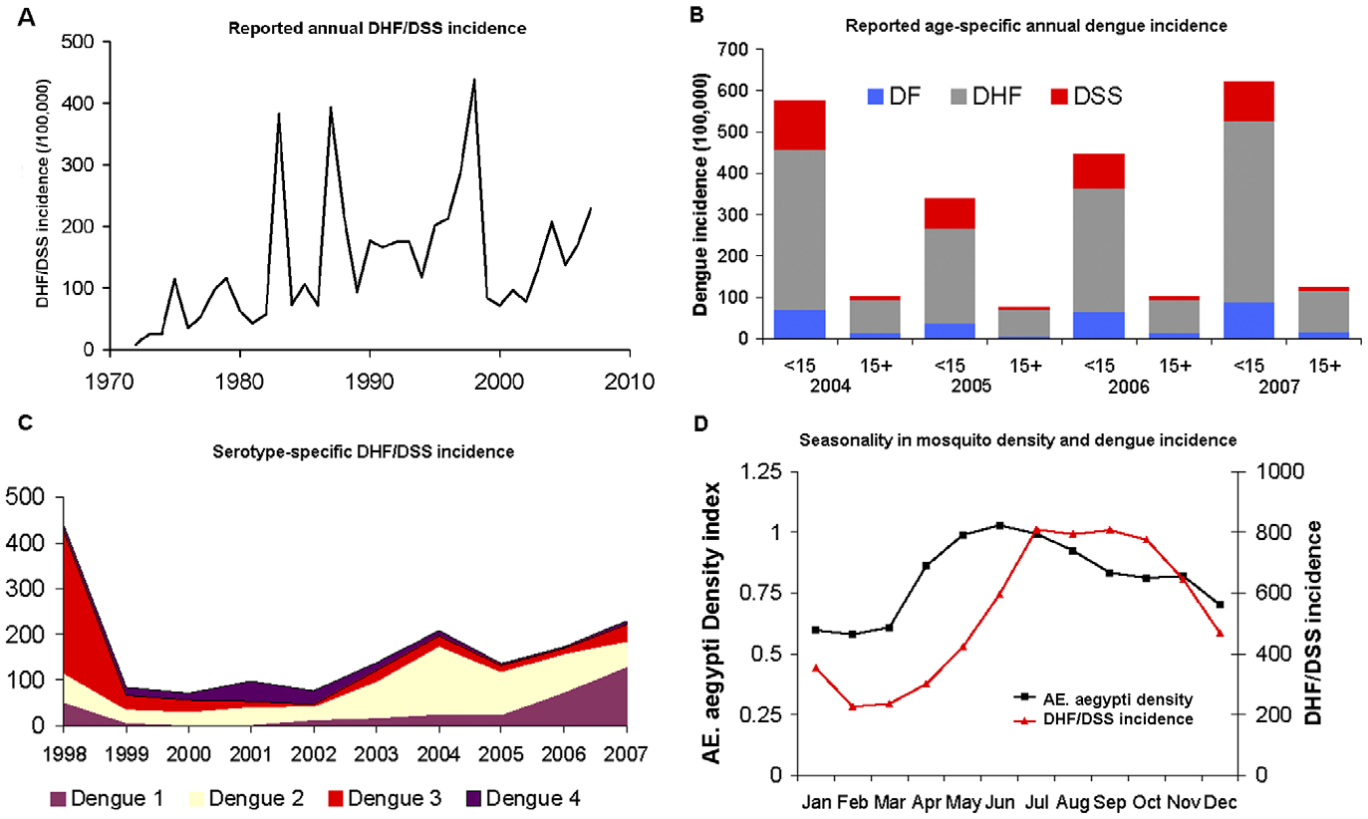
Results of dengue surveillance in Southern Vietnam. a. Reported DHF incidence from 1972 to 2007 (per 100,000 inhabitants). b. Reported dengue incidence for <15 years old and 15+ years old individual per level of severity (per 100,000 inhabitants) c. based on reported incidence and serotype distribution of isolated virus (about 1% of reported cases are subject to virus isolation) d. Seasonality in vector (Aedes Aegypti) density and monthly DHF incidence. Seasonality was assessed through locally weighted scatterplot smoothing [61]. Data derived from the surveillance system of dengue in Southern Vietnam coordinated by the Pasteur Institute in Ho Chi Minh.

The available surveillance data (Figure 2) include: the annual incidence of dengue hemorrhagic fever and dengue shock syndrome (DHF and DSS) for the 36-year period 1972–2008; the monthly and age-specific incidence of DHF/DSS for the period 1998–2007; information on dengue fever (DF) for the 2004–2007 period; and the serotype distribution determined through virus isolation, and the vector density.

**Figure 3:**
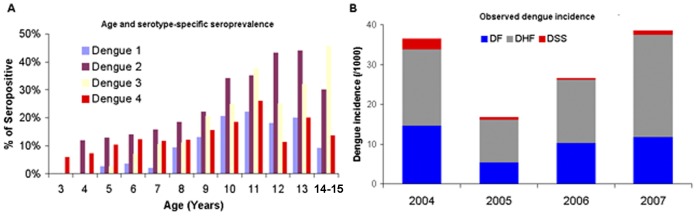
Results of a prospective cohort study in Long Xuen [**34**]. a. Age and serotype specific seroprevalence rate observed in a prospective cohort study performed in An Giang from (2004–2007) b. DF, DHF and DSS incidence observed in 3–15 year-old children.

The rate of dengue infection, which is central to our transmission model, cannot be derived from surveillance data. For this purpose, we used the results of a prospective cohort study conducted in Long Xuyen, the capital of capital of An Giang province in the Mekong Delta (Figure 3) [34]. This study followed a dynamic cohort of 3–15 year-old children from 2004 to 2007 and actively surveyed the occurrence of febrile illness, providing a robust picture of the true incidence of dengue among children in Southern Vietnam. Laboratory confirmation of dengue infection was systematically sought for all suspected cases. Additionally, this study included a seroprevalence assessment (using a microneutralization assay for dengue viruses [35]) of the entire cohort each December, providing an estimate of the evolution of serotype-specific seroprevalence with age. Seroprevalence was seen to increase with age, most notably for serotypes 2 and 3 (Figure 3b). Tien et al. [34] reported that the attack rate in their cohort study was 6 times higher than that reported by routine surveillance system for the same period and age group (respectively 3% and 0.5%). We used the results from this study to derive severity-specific correction factors (15.6 in the case of DF, 5.1 for DHF and 1.1 for DSS) to account for the underreporting of cases inherent to routine surveillance data. In the absence of direct information for adults we used the same correction factor across all ages.

To ensure consistency between the two datasets, cases detected in the cohort study were classified as DF, DHF or DSS according to the case definitions used in the Vietnamese national surveillance program, which are derived from World Health Organization (WHO) guidelines published in 1997 [36]. According to this case definition, severe cases correspond here to DHF/DSS and mild cases to DF. In 2009, the WHO revised their guidelines and definitions of dengue disease [37].

Finally, in addition to dengue surveillance data and the results of this prospective cohort study, we used demographic information and data on the duration of infection in humans and in mosquitoes to populate our model (see Table S1.1 in Text S1).

### Adjustment of Model Parameter Values

We used a two-step process to adjust the model parameters. Firstly we estimated the transmission parameters from seroprevalence data, and the proportion of infections leading to a mild or severe disease from age and severity-specific incidence. We used for this estimation a Maximum Likelihood approach similar to Ferguson et al. [19] and Barraquer et al. [38] combined with an Expectation-Maximization algorithm [39]. This first step, based on the steady state associated with the model, assumed stable dengue dynamics. Using a method similar to Cuong et al. [40] we tested for this stability and detected no significant trend in dengue incidence over the last 30 years in southern Vietnam. Secondly we adjusted the model parameters to reproduce the observed seasonality in vector density and monthly dengue incidence.

**Figure 4:**
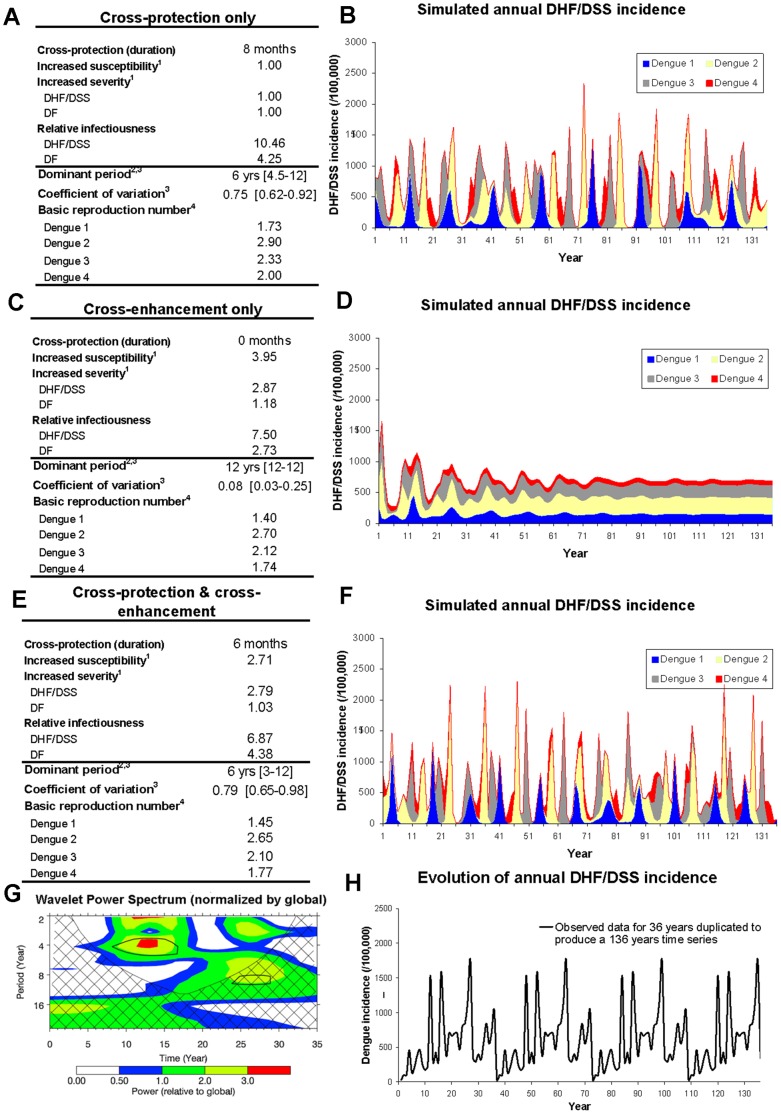
Simulated and observed evolution of annual dengue incidence for different scenarios of serotype interactions: Model simulation with short term cross-protection only (A,B), Model simulation with cross-enhancement only (C,D), Model simulation with cross-protection & cross-enhancement (E,F). Wavelet analysis for observed data (G) and observed annual DHF/DSS incidence corrected for under-reporting (H). Footnotes of panels A, C, E: 1. Increased susceptibility and severity in case of secondary infection 2. Dominant period assessed using Fourier power spectrum over 36 consecutive years, 3. Median, minimum and maximum 4. The basic reproduction number reported is the annual average (average vector density and daily biting rates). In Wavelet analysis for observed data (G), the power has been scaled by the global wavelet spectrum. The cross-hatched region is the cone of influence, where zero padding has reduced the variance. Black contour is the 10% significance level [41]. The 36 years of available data were duplicated to improve comparability with model results (H).

The wavelet power spectrum [41] (Figure 4H) and Fourier power spectrum [42] of the observed DHF/DSS annual incidence highlighted the existence of significant 4–6 year cycles. Reproducing this annual periodicity was not the subject of a direct estimation procedure. Instead we explored whether the observed periodicity could be reproduced qualitatively for different scenarios of immunological interactions between serotypes. Two endpoints were derived for this purpose: the duration of multiannual cycles as measured by the dominant period in the Fourier power spectrum, and the coefficient of variation of the annual incidence (0.73 for the 36 years of available data). We used a Latin hypercube design [43] to explore the space of possible serotype interactions and considered three distinct groups: a group of scenarios based on cross-protection only, a group of scenarios based on cross-enhancement only and a group of scenarios combining cross-protection and cross-enhancement.

### Vaccination

Based on currently available information [6], it was assumed that a fully susceptible individual requires three doses to get a high level of protection against the serotypes included in the vaccine. Protection afforded by vaccination was not considered to be absolute: each vaccinee was assumed to have a residual risk of infection in case of exposure [44]. We account for the limited duration of protection by considering that the vaccine-acquired protection is likely to wane over time. Natural boosting, the impact of which has been analyzed for other infectious diseases [45]–[47], was also accounted for by considering that a bite of a vaccinated subject by an infectious mosquito that do not result into a breakthrough case may increase the level of protection (Figure 1).

We considered a tetravalent vaccine administered in a three-dose, 0–6–12 month regimen in accordance with the regimen being investigated in phase 3 trials [6]. With respect to the protection conferred by the vaccine, we explored levels of initial efficacy ranging from 70% to 90% and duration of high protection ranging from 10 to 30 years. Vaccine seroprotection lasting more than 30 years has for instance been reported for yellow fever vaccines [48]. To remain conservative, we assumed that increased severity induced by cross-enhancement would apply equally after vaccination and natural infection.

We explored a 3-dose routine vaccination program at the ages 12, 18, and 24, and considered that vaccination coverage (for all three doses) was 90% or 70%.

## Results

We focus here on the main insights provided by the model after exploring various scenarios and comparing model outputs with observations. Text S2 provides a detailed presentation of the results of the various scenarios considered.

### Model Calibration and Immunological Interaction between Serotypes

The first calibration step enabled us to rule out as unrealistic several scenarios for which the model outputs were incompatible with the observed average incidence, including permanent cross-protection and high degrees of cross-enhancement (i.e., a >5-fold increase in susceptibility, or >10 increase in the risk of developing symptomatic infection in case of secondary infection). We therefore limited our exploration of interaction scenarios to a plausible range (see S1.5 in Text S1). However this step also showed that a large number of alternative scenarios were equally able to produce the observed average incidence.

The ability to reproduce the observed multiannual cycles is a further distinguishing feature to assess the validity of a scenario. We found that these observations cannot be reproduced in the absence of any form of immunological interaction between serotypes (model S2.5.D). Short term cross-protection against infection (but not against symptomatic disease only, see model S2.5A) was found to be critical to reproducing the observed periodicity (Table S2.1.1–S2.1.3 in Text S2). This type of interaction desynchronizes the oscillations in the serotype-specific incidence, resulting in multiannual cycles comparable to observed data as illustrated Figure 4B. The exact duration of cross-protection was, however, difficult to establish: we identified six models with an average duration for cross-protection ranging from 6 to 17 months, that produced results compatible with observed dynamics.

Models considering cross-enhancement alone, whether temporary or permanent, did not reproduce the observed dengue transmission dynamics as, unlike cross-protection, it could not desynchronize the dynamics of each serotype. Nor could the observed transmission dynamics be reproduced by artificially introducing 1-year interval between the introductions of each serotype in the population: the initial 1-year interval was lost over time in the ‘cross-enhancement alone’ scenarios.

Three models combining cross-protection and cross-enhancement were consistent with observed data. These models showed a higher proportion of cases induced by secondary infections: 58%–60% of DHF/DSS cases were secondary infections (93%–95% in individuals aged ≥15) versus 41%–45% for models only including seroprotection.

We also considered a scenario where the first two infections conferred complete protection against any further dengue infection. This scenario generated longer cycles (18 years) and smaller annual variations (11% [3]–[41]) than observed in southern Vietnam.

In summary the calibration procedure led us to select six scenarios: 3 that only include cross-protection between serotypes (Models S2.2A–S2.2C) and 3 that combined cross-protection and cross-enhancement (Models S2.4A–S2.4C). These six models were used to explore the impact of vaccination programs.

### Demographic Changes

The impact of demographic changes was explored (models 2.6A–B) by considering a sudden change in the population growth rate for both the human and mosquito populations (similar sized change for each population) with the model S2.4A used as a reference. Doubling the population growth rate of both host and vector led to a higher frequency of outbreaks (median duration of outbreak cycles 3 years), a decrease in the average age of dengue cases (14.3 years to 10.5 years) and an increase in dengue incidence (+80%). When population growth was set to zero, which corresponds to an ageing population, the median cycle duration increased from 6 to 12 years, dengue incidence decreased by 50% and the mean age of cases increased to 21.5 years.

### Vector Control

A sudden reduction in the size of the vector population, such as that caused by the implementation of a vector control program, reduces the disease incidence in the short term, but does not alter the long-term dynamics of transmission. For example, a 50% reduction for 2 years (model S2.6E) led to a dramatic, 96% reduction in dengue incidence measured over 5 years. However, the reduction over 10 years was only 20%, and became null over a 15 years period. The reduction in incidence seen in the short term is cancelled out in the long term by a higher incidence after the program’s interruption.

Disassociating the growth rate of the human and vector populations had a much stronger impact in the long run. Stopping the vector population growth, while allowing the human population to continue growing (model S2.6C), eliminated the circulation of dengue after 20 years. Doubling the vector population growth rate on the other hand resulted in more frequent outbreaks and a tripling of the dengue incidence (median duration of cycles 3 years [2.4–3.8]).

### Vaccination

**Figure 5:**
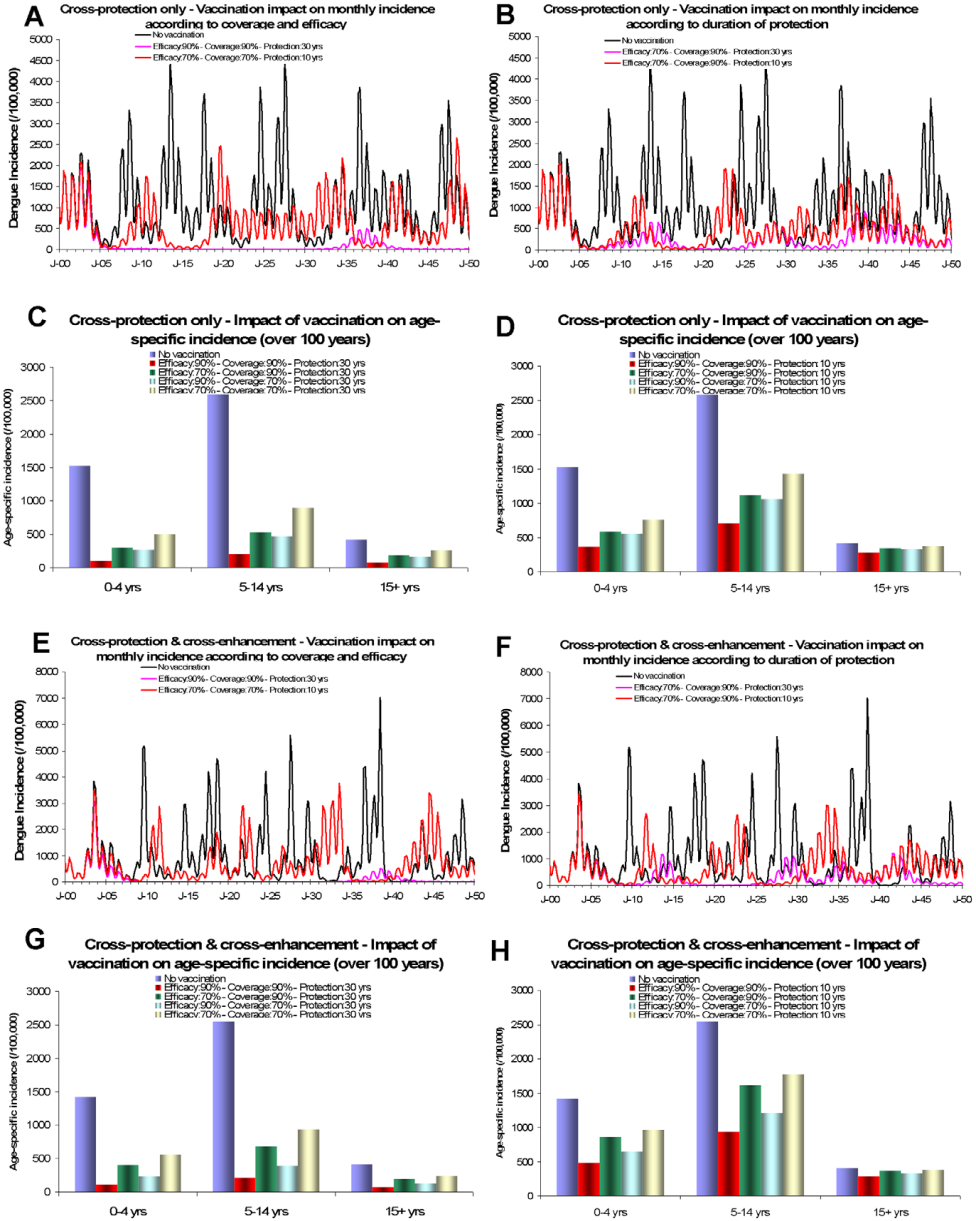
Potential impact of vaccination for a scenario with cross-protection only (A–D) and a scenario combining cross-protection & cross-enhancement (E–H). The two scenarios are those which calibration results are reported Figure 4 (Model S2.2C, S2.4C).

Routine vaccination of 90% of 1 year-olds (Figure 5) with 90% vaccine efficacy after 3 doses and an average duration of full protection of 30 years significantly reduced disease incidence: from 87% to 93% according to the scenario considered over 100 years. Five to ten years were required to observe a marked reduction in the number of cases (14–33% over the first 5 years and 76–85% over the first 25 years). With this scenario, outbreaks become rare and limited in size (Figure 5). Vaccination also increased the mean age of cases (from 14.3 to 19.3–21.3 years).

Due to indirect effects, vaccination benefits both vaccinated and non vaccinated individuals: the reduction of the lifetime risk of infection was respectively 87% and 96% for vaccinated and non vaccinated individuals with models S2.2B, respectively 77% and 90% with model S2.4B.

Decreasing the assumed vaccine efficacy to 70% results in similar changes to the transmission dynamics (Figure 5A–5E) but provided a smaller reduction in disease incidence (a reduction over 100 years ranging from 68% to 80% according to the scenario considered). Larger outbreaks were also observed but remained smaller than without vaccination.

Lowering the vaccination coverage rate from 90% to 70% also results in a smaller reduction in disease incidence: According to the scenario considered, the reduction ranged from 78% to 82% when the vaccine efficacy was 90% and the duration of protection set at 30 years.

Decreasing the assumed average duration of protection from 30 to 10 years had little effect over the first 10 years (Figure 5B–5F) but reduced incidence thereafter. In all scenarios considered, when the duration of protection was set at 10 years and vaccine efficacy at 90%, a 90% vaccination coverage led to a reduction over 100 years ranging from 54% to 70%.

Models including cross-enhancement (S2.4A–C) globally led to a smaller reduction in dengue incidence than models that only considered cross-protection (S2.2A–C). For a vaccine efficacy of 90%, duration of protection of 10 years, and 90% coverage, the overall reduction in incidence was 64–70% for models S2.2A–C and 54–55% for models S2.4A–C. Models including cross-enhancement also led to a slightly lower reduction of severe cases than mild cases (respectively 51–52% and 58–62%).

Differences between models S2.2A–C and S2.4A–C were more pronounced when efficacy and coverage were set at 70% and duration of protection at 10 years. In this case, dengue incidence was reduced by 38%–44% and 24%–25%, respectively. Conversely, very limited differences were observed when the average duration of full protection is set at 30 years and efficacy and coverage at 90%: respectively 90–93% for models S2.2A–C and 87–90% for models S2.4A–C.

Finally, we also analyzed the impact of demographic changes on vaccination benefits. If the absolute benefit of vaccination is directly affected by a demographic change that impacts dengue incidence, the relative benefit remains relatively stable. For instance, if the average duration of full protection is set at 30 years and efficacy and coverage at 70%, dengue incidence is reduced by 57% if the population grow at a high rate (model S2.6A), by 54% if the population stop growing (model S2.6B) or if the population continue growing at the rate currently observed (model S2.4C).

## Discussion

We built a model capturing most of the features likely to influence the dynamics of dengue transmission and, after validating it against epidemiological data from Southern Vietnam, tested different hypotheses for their ability to reproduce the observed epidemiology of dengue in this highly endemic area. Our results highlight the critical role of short-term cross-protection that desynchronizes the epidemic cycles of the four dengue serotypes, resulting in 5–6 year-cycles consistent with those observed in Southern Vietnam. Wearing and Rohani reached the same conclusion after comparing a model with Thai data [13]. In a recent analysis, Endy et al. [49] also pointed out the importance of cross-protection to explain the results observed in a cohort of Thai children.

Our model suggests that the duration of cross-protection is in the range 6–17 months but did not point to a specific duration. However, our model assumption that this duration follows an exponential distribution may be inaccurate since this implies large variation on the duration of cross-protection from one individual to another. As dengue is a seasonal disease in southern Vietnam, it is plausible that cross-protection of shorter duration, associated with a narrower temporal distribution would suffice to desynchronize epidemics. Cross-protection against disease symptoms alone was insufficient to reproduce the observed multiannual cycles but this result was obtained for a scenario in which asymptomatic infections significantly contribute to disease transmission.

In our model, cross-enhancement without cross-protection did not reproduce the observed epidemiology of dengue in Southern Vietnam. This, however, does not invalidate the existence of immunological cross-enhancement that has been considered in many dengue transmission models [9], [10], [12], [13], [15]–[22]. Indeed, several models combining cross-protection and cross-enhancement were able to reproduce dengue dynamics. More importantly, adding cross-enhancement increased the proportion of symptomatic cases caused by secondary infections to a value (59–74% overall, 85–91% for 15+ years cases), comparable with that reported by Phuong et al for Southern Vietnam (84%) [50]. The model used to assess the impact of vaccination in the presence of cross-enhancement was also characterized by a higher proportion of severe cases among secondary infections than among primary infections (70% versus 60%). This higher proportion has been argued to indicate the existence of antibody-dependent enhancement [51].

These analyses also provide insights into other aspects of the transmission dynamics of dengue. Consistent with findings reported by Wikramaratna et al. [52] our model provided a better fit to observations under the assumption that individuals can become sequentially infected by all four serotypes, rather than when two infections were assumed to provided complete protection. Among demographic factors, we notably observed that a decrease in birth rate increased the average age of dengue cases and increased the interval between major outbreaks. The same pattern was identified by Cummings et al. [24] as the main explanation of the recent evolution of dengue epidemiology in Thailand.

By exploring the effect of the relative size of the human and vector populations, we saw that temporarily reducing the size of the vector population had only a short term effect of the transmission dynamics. We equate this with the impact of temporary vector control activities, some of which have had short term success but have not succeeded in maintaining disease reductions in the long run [53]. The results obtained indicate that any interruption (or reduction) in vector control may counteract previous health benefits. The situation in Latin America where dengue reemerged in the seventies after being certified free of dengue between 1952 and 1965 provides a good illustration of this phenomenon [54]. Dengue resurgence in Singapore after 2 decades of successful control is another example of the difficulties of controlling dengue through vector control [55].

Our model was sensitive to the disassociation of the growth rate of the human and vector populations. This can be equated with lifestyle or environmental changes, such as urbanization or global warming, which have consequences on the ecology of the vector population and thereby on dengue transmission [56]. Although our model reproduces some general trends regarding the impact of vector control activities and environmental change, it is however important to stress that it is clearly not adapted to a detailed analysis of these phenomena.

Our analysis was based on two complementary sources of information: routine surveillance in Southern Vietnam and a prospective cohort study with active surveillance conducted in the area [34]. Routine surveillance systems can provide a critical general overview of epidemiology and its evolution over extended periods. In contrast, prospective studies can provide a more accurate estimation of epidemiology over a short period, including estimations for serotype-specific rate of infection, such as those used in our model. Furthermore, when feasible, surveillance data and data collected prospectively, such as that reported by Tien et al. [34], can be compared to estimate the level of under-reporting to the surveillance system. Using this approach, Tien et al. estimated that the actual number of dengue cases was 6 times higher than that reported to the surveillance system and that severe cases are also more likely to be reported. Similar findings were reported from Thailand [57], [58].

There are, however, some limitations to the combined use of these data sources. The data collection period differed between the two sources (1972–2007 for the routine surveillance data 2004–2007 for the prospective cohort study, see Text S1), as did the data catchment area (the surveillance system covered the entire south of Vietnam, whereas the study covered the Long Xuyen area only), and the age range (restricted to 3–15 years in the prospective study). Other possible limitations include the accuracy of dengue serological tests [35], [58] and the quality of surveillance, which evolves with time [34]. This notably led us, in the absence of direct information for adults, to use the same expansion factor across all ages and it is plausible that the level of under-reporting varies according to age.

We did not identify one single scenario of cross-interactions. This can be seen as a limitation of our analysis. Considering the heterogeneous nature of data available to us and the complex nature of dengue dynamics, we in fact deliberately chose to assess the impact of vaccination for a range of plausible scenarios of interactions between serotypes. A more direct estimation of the duration of cross-protection or of the characteristics of cross-enhancement using recent advances in statistical methods for transmission models [59], [60] is however an important direction for future research. Although the tests we performed indicated that this assumption is reasonable, another possible limitation of the analysis performed is our consideration in the calibration procedure that dengue epidemiology is globally stable in Southern Vietnam despite large interannual variations. A direct consideration of epidemiological or demographic changes in the calibration procedure could also be explored in future research.

Assessing the potential impact of vaccination was an important motivation behind the development of this model. Results suggest that vaccination will not only reduce disease incidence but will also modify the transmission dynamics. Indeed, vaccination was seen to decrease the frequency and magnitude of outbreaks, and to alter the age distribution of cases. The overall impact of vaccination as indicated by our model depends not only on vaccine efficacy, but also on the duration of protection conferred and on the level of vaccination coverage.

In contrast to the findings discussed above, that may be applicable to a range of vaccine-preventable disease, cross-enhancement is more specific to the immunopathology of dengue. Vaccination was shown to have a greater impact in scenarios that only considered short term cross-protection, but the difference between these scenarios and those combining cross-protection and cross-enhancement is limited in most cases. It should be noted that this finding was based on the assumption that cross-enhancement would apply equally after vaccination and natural infection. This is a conservative assumption as none of the preclinical or clinical data accumulated to date support the hypothesis that vaccination can lead to enhanced disease [6], [62].

In conclusion, we developed a model capable of reproducing the epidemiology of dengue in Southern Vietnam to provide a theoretical framework for future studies on dengue prevention and vaccination. Our results highlight the importance of short-term cross-protection in the transmission dynamics of these viruses. In addition to vaccination results presented here, this model could readily be expanded to consider for example: the optimum age for vaccination; the value of combining vaccination with other prevention measures; the way epidemiological or demographic changes affect the impact of vaccination, or the relative short and long-term benefits of various strategies based on routine and/or mass vaccination campaigns.

## Supporting Information

Text S1 **Supplementary technical information.**
(DOC)

Text S2 **Supplementary results.**
(DOC)
