# Supplementary material for: Transmission Dynamics of the Four Dengue Serotypes in Southern Vietnam and the Potential Impact of Vaccination
Source: PLoS One. 2012 Dec 10;7(12):e51244. doi: 10.1371/journal.pone.0051244 (PMC3519629; doi:10.1371/journal.pone.0051244)
Supplement: Text S1 — Supplementary technical information. (DOC) [file pone.0051244.s001.doc]

# Transmission dynamics of the four dengue serotypes in Southern Vietnam and the potential impact of vaccination

# Text S1 - Supplementary technical information

[S1.1 Differential equations defining the model 2](#__RefHeading___Toc334258463)

[S1.1.1 Host population 2](#__RefHeading___Toc334258464)

[S1.1.1.1 The infection process 3](#__RefHeading___Toc334258465)

[S1.1.1.2 The ageing and vaccination process 4](#__RefHeading___Toc334258466)

[S1.1.1.3 Force of infection 6](#__RefHeading___Toc334258467)

[S1.1.2 Vector population 6](#__RefHeading___Toc334258468)

[S1.2 Steady state 8](#__RefHeading___Toc334258469)

[S1.2.1 Host population 8](#__RefHeading___Toc334258470)

[S1.2.2 Vector population 10](#__RefHeading___Toc334258471)

[S1.2.3 Basic reproduction number 10](#__RefHeading___Toc334258472)

[S1.3 Model Inputs 11](#__RefHeading___Toc334258473)

[S1.3.1 Dengue infection process 11](#__RefHeading___Toc334258474)

[S1.3.2 Demographic parameters 11](#__RefHeading___Toc334258475)

[S1.3.3 Epidemiology of dengue in Southern Vietnam. 12](#__RefHeading___Toc334258476)

[S1.4 Calibration procedure 13](#__RefHeading___Toc334258477)

[S1.4.1 Estimating the transmission parameters and proportion of infections leading to a symptomatic disease 14](#__RefHeading___Toc334258478)

[S1.4.2 Calibration of vector birth and biting rates 15](#__RefHeading___Toc334258479)

[S1.4.3 Comparison of model dynamics with observed annual dynamics 15](#__RefHeading___Toc334258480)

[S1.5 Latin hypercube design to explore serotype interaction scenarios 16](#__RefHeading___Toc334258481)

[S1.6 Evolution of annual DHF/DSS incidence 17](#__RefHeading___Toc334258482)

# Differential equations defining the model

## Host population

Individuals are characterized by their age group and status for each of the 4 dengue serotypes, represented as $H_{i}^{jklm}(t)$, where t is the date, subscript number indicates the age group, and superscript numbers represent the immune/disease status for each serotype. 10 different states were considered:

1. Susceptible
2. Latent stage or incubation period of a dengue infection
3. Infectious stage of a severe dengue infection
4. Infectious stage of a mild dengue infection
5. Infectious stage of an asymptomatic dengue infection
6. Short-term immunity (with possibility of cross-protection or cross-enhancement for infection with other serotypes)
7. Long-term immunity (with possibility of cross-protection or cross-enhancement for infection with other serotypes)
8. Low protection against infection due to vaccination
9. Medium protection against infection due to vaccination
10. High protection against infection due to vaccination

The set of differential equations that define the model provide a representation of the infection, ageing, and vaccination processes and is given by:

$$\frac{dH_{i}^{jklm}(t)}{dt}=I_{1,i}^{jklm}(t)+I_{2,i}^{jklm}(t)+I_{3,i}^{jklm}(t)+I_{4,i}^{jklm}(t)+D_{i}^{jklm}(t)$$

Where $I_{1,i}^{jklm}(t),I_{2,i}^{jklm}(t),I_{3,i}^{jklm}(t),I_{4,i}^{jklm}(t)$ correspond to the infection processes for serotypes 1, 2, 3 and 4, and $D_{i}^{jklm}(t)$ to the ageing and vaccination process.

### The infection process

The infection process is identical whatever the serotype. For simplicity, we only present this process for serotype 1 ($I_{1,i}^{jklm}(t)$).

$$\begin{matrix} \forall\text{i}\in\left\{1,..,N\right\} ,\forall\text{k}\in\left\{1,..,9\right\} , \forall\text{l}\in\left\{1,..,9\right\} , \forall\text{m}\in\left\{1,..,9\right\} \\ \left\{\begin{matrix} I_{1,i}^{0klm}(t)=-su_{1}^{klm}\lambda_{i}^{1}(t)H_{i}^{1klm}(t) \\ I_{1,i}^{1klm}(t)=su_{1}^{klm}\lambda_{i}^{1}(t)\left[H_{i}^{0klm}(t)+(1-ve_{^{l}}^{1})H_{i}^{7klm}(t)+(1-ve_{^{m}}^{1})H_{i}^{8klm}(t)+(1-ve_{^{h}}^{1})H_{i}^{9klm}(t)\right]-\tau_{H}H_{i}^{1klm}(t) \\ I_{1,i}^{2klm}(t)=es_{1}^{klm}sev_{_{i}}^{1}\tau_{H}H_{i}^{1klm}(t)-\nu_{H}H_{i}^{2klm}(t) \\ I_{1,i}^{3klm}(t)=em_{1}^{klm}mil_{_{i}}^{1}\tau_{H}H_{i}^{1klm}(t)-\nu_{H}H_{i}^{3klm}(t) \\ I_{1,i}^{4klm}(t)=\left[1-es_{1}^{klm}sev_{_{i}}^{1}-em_{1}^{klm}mil_{_{i}}^{1}\right]\tau_{H}H_{i}^{1klm}(t)-\nu_{H}H_{i}^{4klm}(t) \\ I_{1,i}^{5klm}(t)=\nu_{H}\left(H_{i}^{2klm}(t)+H_{i}^{3klm}(t)+H_{i}^{4klm}(t)\right)-\varepsilon_{H}H_{i}^{5klm}(t) \\ I_{1,i}^{6klm}(t)=\varepsilon_{H}H_{i}^{5klm}(t) \\ I_{1,i}^{7klm}(t)=w^{1}H_{i}^{8klm}(t)-su_{1}^{klm}\lambda_{i}^{1}(t)H_{i}^{7klm}(t) \\ I_{1,i}^{8klm}(t)=w^{1}H_{i}^{9klm}(t)-\left(w^{1}+su_{1}^{klm}\lambda_{i}^{1}(t)\right)H_{i}^{9klm}(t) \\ I_{1,i}^{9klm}(t)=su_{1}^{klm}\lambda_{i}^{1}(t)\left[ve_{^{l}}^{1}H_{i}^{7klm}(t)+ve_{^{m}}^{1}H_{i}^{8klm}(t)-(1-ve_{^{h}}^{1})H_{i}^{9klm}(t)\right] -w^{1}H_{i}^{9klm}(t) \end{matrix}\right. \end{matrix}$$

Where $\lambda_{i}^{1}(t)$ is the force of infection for serotype 1 in age class i; *τ_H_*, *ν_H_, ε_H_*  are the time-independent transition rates between immune/disease states, and $sev_{_{i}}^{s},mil_{_{i}}^{s}$ represent the risk that individuals develop severe or mild dengue disease respectively $es_{1}^{klm}sev_{_{i}}^{1}+em_{1}^{klm}mil_{_{i}}^{1}\leq1$.

The terms $ve_{l}^{1},ve_{m}^{1},ve_{h}^{1}$ respectively represent low, medium and high levels of protection. When exposed to the virus, a vaccinated subject can either develop an infection or benefit from a boosting effect which increases their protection

The term$w^{1}$ is the rate at which vaccine-acquired protection against serotype 1 wanes over time.

Two categories of serotype interactions are considered in the model:

1. A modified probability of infection:

$$su_{j}^{klm}=\mathop{Min}\limits_{x\in\left\{k,l,m\right\}} \kappa_{x}^{1}$$

$\kappa_{x}^{1}$ is the level of susceptibility to serotype 1 induced by status *x*. The value of $su_{1}^{klm}$ indicates either cross-protection ($0<su_{1}^{klm}<1$) or cross-enhancement ($su_{1}^{klm}>1$)

1. A modified risk of developing a severe ($es_{1}^{klm}$) or mild ($em_{1}^{klm}$) dengue disease:

$es_{1}^{klm}=\left\{\begin{matrix} \mathop{Max}\limits_{x\in\left\{k,l,m\right\}} s_{x}^{1} & , & s_{k}^{1}>1\cap s_{l}^{1}>1\cap s_{m}^{1}>1 \\ \mathop{Min}\limits_{x\in\left\{k,l,m\right\}} s_{x}^{1} & , & s_{k}^{1}<1\cup s_{l}^{1}<1\cup s_{m}^{1}<1 \end{matrix}\right.$,$em_{1}^{klm}=\left\{\begin{matrix} \mathop{Max}\limits_{x\in\left\{k,l,m\right\}} m_{x}^{1} & , & m_{k}^{1}>1\cap m_{l}^{1}>1\cap m_{m}^{1}>1 \\ \mathop{Min}\limits_{x\in\left\{k,l,m\right\}} m_{x}^{1} & , & m_{k}^{1}<1\cup m_{l}^{1}<1\cup m_{m}^{1}<1 \end{matrix}\right.$

$s_{x}^{1}, m_{x}^{1}$ correspond respectively to the relative risk of developing severe or mild heterotypic disease for subjects in state *x* ($s_{x}^{1}\geq0 , m_{x}^{1}\geq0$).$es_{1}^{klm}$ and $em_{1}^{klm}$ indicates either cross-protection ($0<es_{1}^{klm}<1 , 0<em_{1}^{klm}<1$) or cross-enhancement ($es_{1}^{klm}>1 , em_{1}^{klm}>1$)

### The ageing and vaccination process

We considered a population with a constant age structure and a continuous flow of individuals between age groups [1]. Vaccination occurs when individuals transit between age groups (vaccination coverage at time t in age group i is given by$vc_{i}(t)$)

Unless vaccinated at birth, newborns are susceptible to infection such that:

$$\left\{\begin{matrix} \frac{D_{1}^{0000}(t)}{dt}=(1-vc_{1}(t))B_{H}(t)-\left(A_{1}+\mu_{1}\right)H_{1}^{0000}(t) & , & \text{jklm}=0000 \\ \frac{D_{1}^{7777}(t)}{dt}=vc_{1}(t)B_{H}(t)-\left(A_{1}+\mu_{1}\right)H_{1}^{0000}(t) & , & \text{jklm}=7777 \\ \frac{D_{1}^{jklm}(t)}{dt}=-\left(A_{1}+\mu_{1}\right)H_{1}^{jklm}(t) & , & \text{jklm}\notin\left\{\text{0000,7777}\right\} \end{matrix}\right.$$

Where *μ_1_* is the mortality rate in the first age group and *A_1_* is the transition rate between age groups and is defined as:

$$A_{i}=\frac{\mu_{i}+q}{e^{-\left(\mu_{i}+q\right)l_{i}}-1}$$

Where *q* is the population growth rate and *l_i_* is the breadth of age class i.

The number of births is linked to the size of the population through the equation

$$B_{H}(t)=f_{H}*\sum_{i} \sum_{j,k,l,m} H_{i}^{jklm}(t)=f_{H}*\sum_{i} P_{i}(t)$$

Where $f_{H}$ corresponds to the birth rate and $P_{i}(t)$ the number of individuals in age class i. At demographic equilibrium (i.e. stable age structure), the following equations hold:

$$\forall i>1 , P_{i}(t)=\frac{A_{i-1}P_{i-1}(t)}{A_{i}+\mu_{i}+q} ; P_{1}(t)=\frac{B_{H}(t)}{A_{1}+\mu_{1}+q} ,f_{H}=\frac{P_{1}(t)\left(A_{1}+\mu_{1}+q\right)}{\sum_{i} P_{i}(t)}$$

For subsequent age groups, the ageing and vaccination process becomes:

$$\frac{D_{i}^{jklm}(t)}{dt}=A_{i-1}\left(vc_{i}(t)\sum_{\begin{matrix} p=0,9 q=0,9 \\ r=0,9 s=0,9 \end{matrix}} \left[\varphi_{jp}\varphi_{kq}\varphi_{lr}\varphi_{ms}H_{i-1}^{pqrs}(t)\right] +\left(1-vc_{i}(t)\right)H_{i-1}^{jklm}(t)\right)-\left(A_{i}+\mu_{i}\right)H_{i}^{jklm}(t)$$

$\varphi_{jp},\varphi_{kq},\varphi_{lr},\varphi_{ms}$ are binary variables defining the impact of vaccination according to the individual’s dengue status. They can be viewed as the elements of the matrix Φ defined as follows:

$$\Phi=\left[\begin{matrix} 0 & 0 & 0 & 0 & 0 & 0 & 0 & 1 & 0 & 0 \\ 0 & 1 & 0 & 0 & 0 & 0 & 0 & 0 & 0 & 0 \\ 0 & 0 & 1 & 0 & 0 & 0 & 0 & 0 & 0 & 0 \\ 0 & 0 & 0 & 1 & 0 & 0 & 0 & 0 & 0 & 0 \\ 0 & 0 & 0 & 0 & 1 & 0 & 0 & 0 & 0 & 0 \\ 0 & 0 & 0 & 0 & 0 & 1 & 0 & 0 & 0 & 0 \\ 0 & 0 & 0 & 0 & 0 & 0 & 1 & 0 & 0 & 0 \\ 0 & 0 & 0 & 0 & 0 & 0 & 0 & 0 & 1 & 0 \\ 0 & 0 & 0 & 0 & 0 & 0 & 0 & 0 & 0 & 1 \\ 0 & 0 & 0 & 0 & 0 & 0 & 0 & 0 & 0 & 1 \end{matrix}\right]$$

For each serotype, the representation of the vaccination process is used to describe a situation where 3 vaccination doses are required to achieve a high level of protection.

### Force of infection

The force of infection for humans in age group i due to serotype 1,2,3,4 is given by:

$$\lambda_{i}^{s}(t)=\frac{\beta_{H}^{s}\delta_{i}\alpha_{V}(t)}{\sum_{i} \delta_{i}N_{i}(t)}YV^{s}(t) , s\in\left\{1,2,3,4\right\}$$

Where *N_i_(t)* is the number of individuals in age group i, $\beta_{H}^{s}$ the probability that the bite will result in infection and *δ_i_* the relative risk of being bitten for an individual in age group i. $\alpha_{V}(t)$ is the time-dependant biting rate per infectious vector.

## Vector population

The dengue status of the vector population is defined through 9 state variables whose value is given by the following equations:

$$\begin{matrix} \frac{dV(t)}{dt}=B_{V}(t)-\left(\sum_{s} \theta^{s}(t)+\mu_{V}\right)V(t) \\ \forall\text{s}\in\left\{\text{1,2,3,4}\right\} \\ \left\{\begin{matrix} \frac{dEV^{s}(t)}{dt}=\theta^{s}(t-d)V_{j}(t-d)e^{-\mu_{V}d}-\left(\mu_{V}+\tau_{V}\right)EV^{s}(t) \\ \frac{dYV^{s}(t)}{dt}=\tau_{V}EV^{s}(t)-\mu_{V}YV^{s}(t) \end{matrix}\right. \end{matrix}$$

*V(t)* represents susceptible vectors, $EV^{s}(t)$ vectors in a latent state (i.e. incubation period) due to an infection with serotype s and $YV^{s}(t)$ vectors infectious for serotype s.

*μ_v_*  is the death rate in the vector population and *τ_v_* the transition rate between the latent and infectious state. *d* corresponds to the minimum duration a vector remains in a latent stage before becoming infectious. The force of infection is given by $\theta^{s}(t)$*.*

The number of new adult female mosquitoes in the population is defined by

$$B_{V}(t)=f_{V}(t)*\left(V(t)+\sum_{s} \left[EV^{s}(t)+YV^{s}(t)\right]\right)$$

Where $f_{V}(t)$ is the time-varying number of new adult female mosquitoes generated by each adult female mosquito.

The death rate of the vector population was calculated using information on life expectancy (LE_V_) and maximum lifetime (*l_v_*) as follows :

$$\begin{matrix} \mu_{V}=\mu_{V}^{0}+A_{V} \\ \left\{\mu_{V}^{0}:LE_{V}=\left(1-e^{-\mu_{V}^{0}}\right)/\mu_{V}^{0}\right\},A_{V}=\frac{\mu_{V}^{0}+q}{e^{-(\mu_{i}+q)l_{v}}-1} \end{matrix}$$

The serotype-specific force of infection for vectors is given by:

$$\left\{\left.\begin{matrix} \theta^{1}(t)=\frac{\beta_{V}\alpha_{V}(t)}{\sum_{i} \delta_{i}N_{i}(t)}\sum_{i} \left(\delta_{i}\sum_{k,l,m} \left(\eta_{1}^{1}H_{i}^{3klm}(t)+\eta_{2}^{1}H_{i}^{4klm}(t)+\eta_{3}^{1}H_{i}^{5klm}(t)\right)\right) \\ \theta^{2}(t)=\frac{\beta_{V}\alpha_{V}(t)}{\sum_{i} \delta_{i}N_{i}(t)}\sum_{i} \left(\delta_{i}\sum_{j,l,m} \left(\eta_{s}^{2}H_{i}^{j3lm}(t)+\eta_{m}^{2}H_{i}^{j4lm}(t)+\eta_{a}^{2}H_{i}^{j5lm}(t)\right)\right) \\ \theta^{3}(t)=\frac{\beta_{V}\alpha_{V}(t)}{\sum_{i} \delta_{i}N_{i}(t)}\sum_{i} \left(\delta_{i}\sum_{j,k,m} \left(\eta_{s}^{3}H_{i}^{jk3m}(t)+\eta_{m}^{3}H_{i}^{jk4m}(t)+\eta_{a}^{3}H_{i}^{jk5m}(t)\right)\right) \\ \theta^{4}(t)=\frac{\beta_{V}\alpha_{V}(t)}{\sum_{i} \delta_{i}N_{i}(t)}\sum_{i} \left(\delta_{i}\sum_{j,k,l} \left(\eta_{s}^{4}H_{i}^{jkl3}(t)+\eta_{m}^{4}H_{i}^{jkl4}(t)+\eta_{a}^{4}H_{i}^{jkl5}(t)\right)\right) \end{matrix}\right|\right.$$

Where $\eta_{s}^{1},\eta_{m}^{1},\eta_{a}^{1}$ is the level of infectiousness of severe, mild and asymptomatic serotype 1 infections. β_v_ is the transmission probability from host to vector and $\alpha_{V}(t)$ the time-dependant biting rate.

This model is coded in the C# language for .NET framework 4 and is solved numerically using Runge-Kutta methods.

# Steady state

Steady state is obtained by solving the following equations:

$$\frac{dH_{i}^{jklm}(t)}{dt}=0, \frac{dV(t)}{dt}=0, \frac{dEV^{s}(t)}{dt}=0, \frac{dYV^{s}(t)}{dt}=0$$

## Host population

The solution for hosts ($\bar{H}_{i}^{jklm}(t)$) requires first to define the numerators and denominators related to the infection process ($n_{1,i}^{jklm}(t) , d_{1,i}^{jklm}$):

Using$n_{s,i}^{jklm}(t) , d_{s,i}^{jklm}(t)$, the solution for hosts can therefore be written as:

The average force of infection corresponds in these equations to$\bar{\lambda}_{i}^{s}$,

The incidence of severe and mild cases $dsev_{i}^{s} , dmil_{i}^{s}$for each serotype and age group are given by:

$$\begin{matrix} dsev_{i}^{1}=\sum_{klm} n_{1,i}^{2klm}(t)/P_{i}(t) & , & dmil_{i}^{1}=\sum_{klm} n_{1,i}^{3klm}(t)/P_{i}(t) \\ dsev_{i}^{2}=\sum_{jlm} n_{2,i}^{j2lm}(t)/P_{i}(t) & , & dmil_{i}^{2}=\sum_{jlm} n_{2,i}^{j3lm}(t)/P_{i}(t) \\ dsev_{i}^{3}=\sum_{jkm} n_{3,i}^{jk2m}(t)/P_{i}(t) & , & dmil_{i}^{3}=\sum_{jkm} n_{3,i}^{jk3m}(t)/P_{i}(t) \\ dsev_{i}^{4}=\sum_{jkl} n_{4,i}^{jkl2}(t)/P_{i}(t) & , & dmil_{i}^{4}=\sum_{jkl} n_{4,i}^{jkl3}(t)/P_{i}(t) \end{matrix}$$

## Vector population

The analytical solution of the steady state situation for vectors is given by:

## Basic reproduction number

The steady state provides a means to calculate the basic reproduction number (R_0_). We use a formula similar to the one derived by Ross & McDonald [2] adapted to this model. The serotype-R_0_ ($R_{0}^{s}$) is the product of the average number of infected humans in a susceptible population ($R_{VH}^{s}$) and the average number of infected mosquitoes b ($R_{HV}^{s}$):

$$R_{VH}^{1}=\beta_{H}^{1}\bar{\alpha}_{V}\frac{\tau_{v}e^{-d}}{\left(\tau_{v}+\mu_{v}\right)\mu_{v}}$$

$$R_{0}^{1}=R_{HV}^{1}R_{VH}^{1}$$

# Model Inputs

## Dengue infection process

Table S1.1. Parameters’ values drawn from Bartley et al. [3]

| **Parameter** | **Symbol** | **Value** |
| --- | --- | --- |
| Duration of latent period in host | *1/τ_H_* | 5 days |
| Duration of infectious period in host | *1/ν_H_* | 4.5 days |
| Minimum duration of the latent stage among vectors | *d* | 8 days |
| Average duration of the latent period among vectors | *d*+1/*τ_v_* | 12 days |
| Transmission probability from host to vector | β_v_ | 0.75 |
| Average biting rate of vectors | $\bar{\alpha}_{V}$ | 0.67/day |
| Mean life expectancy of vectors | μ_v_ | 7.7 days |

## Demographic parameters

Table S1.2. Demographic parameters

| **Parameter** | **Symbol** | **Value** |
| --- | --- | --- |
| Age-specific death rates derived from life table^1^ | *μ_i_* |  |
| Age distribution and total population size (2007) ^2^ | $\sum_{i} P_{i}(t)$ |  |
| 0-4 yrs |  | 2,469,544 |
| 5-9 yrs |  | 2,522,390 |
| 5-9 yrs |  | 3,293,940 |
| 10-14 yrs |  | 3,303,416 |
| 15-19 yrs |  | 5,680,752 |
| 20-29 yrs |  | 4,711,668 |
| 30-39 yrs |  | 5,906,714 |
| 40-59 yrs |  | 2,419,978 |
| 60+ yrs |  | 2,469,544 |
| Total |  | 30,308,400 |
| Population growth rate (2000-2007)^2^ | *q* | 1.7% |

1. WHO - Vietnam - both sexes – 2000 (<http://www.who.int/healthinfo/statistics/mortality_life_tables/en/>)

2. General statistics of Vietnam – Mekong River Delta and South East provinces (<http://www.gso.gov.vn/>)

According to available data, the population increased steadily over the last decade in Southern Vietnam (growth rate for 1995-2000, 2000-2005 and 2000-2005: 1.6%, 1.7%, 1.7%) and the life expectancy increased slightly (70.2 in 2000 and 72.2 in 2009).

## Epidemiology of dengue in Southern Vietnam.

Two information sources were used :

1. Regional dengue surveillance data coordinated by the Pasteur Institute in Ho Chi Minh City:
   1. Monthly DHF/DSS incidence data from 1999 to 2007
   2. Reported DF, DHF and DSS incidence (per 100,000 inhabitants) for 2 age groups (<15 years and 15+ years) between 2004 and 2007.
   3. Annual DHF incidence from 1972 to 2007 (per 100,000 inhabitants) used for assessing the periodicity in dengue incidence.
   4. Results of virus isolation for 1998-2007 (about 1% of reported cases are subject to virus isolation).
   5. Monthly variations in vector density measured using the Breteau index (number of positive containers per 100 houses inspected) between 1997 and 2006. These data indicate monthly variation in vector density but do not directly inform on the number of mosquitoes. Based on observed data [4], we considered an average annual ratio of 2 female adult mosquitoes per host to assess the size of the vector population.
2. A prospective cohort study [5] implemented in the An Giang province (Long Xuen). This prospective study provided:
3. Seroprevalence data for 3-15 years old children observed between 2004 and 2007. Dengue seropositivity was assessed using a microneutralization assay that detects antibodies specific to each serotype [6].
4. An estimation of dengue incidence between 2004 and 2007 for the cohort of 3-15 years old children included in the study. These incidence data, based on active surveillance and systematic dengue confirmation enables the level of under-reporting in routine surveillance to be estimated.

These data are presented in Figure 2 and Figure 3.

# Calibration procedure

The calibration procedure was divided into two steps:

1. The first step provides an estimation of the transmission parameters ($\beta_{H}^{S},\delta_{i}$,) and the proportion of infections leading to symptomatic disease ($sev_{_{i}}^{s},mil_{_{i}}^{s}$).
2. The second step allows the calibration of month-specific birth ($f_{V}(t)$) and biting rates ($\alpha_{V}(t)$).

This calibration procedure can be viewed as an adaptation of the usual approach for calibrating the transmission parameters from age-specific seroprevalence data [7,8]

After the calibration of a given scenario of serotype interactions, we determined the relevance of that scenario by comparing the annual dengue dynamics simulated by the model with those dengue dynamics observed in Southern Vietnam from 1972 to 2007.

## Estimating the transmission parameters and proportion of infections leading to a symptomatic disease

The transmission parameters were estimated with age and serotype-specific seroprevalence data [5]. Such as Ferguson et al. [9] and Rodriguez-Barraquer et al. [10], we used maximum likelihood. The multinomial log-likelihood (ignoring the constant) is given by:

$$l(\beta_{H}^{s},\delta_{i})=\sum_{i=1}^{N} \sum_{j=0}^{1} \sum_{j=k}^{1} \sum_{l=0}^{1} \sum_{m=0}^{1} \left(y_{i}^{jklm}\ln\left[S_{i}^{jklm}\right]\right)$$

Where $y_{i}^{jklm}$ is the observed number of subjects of age i for each of the possible seropositive states (0: seronegative, 1: seropositive). $S_{i}^{jklm}$ is the proportion calculated with the model in a steady state situation given $\beta_{H}^{s},\delta_{i}$ values.

After estimating the transmission parameters, the proportion of infections leading to mild or severe disease ($sev_{_{i}}^{s},mil_{_{i}}^{s}$) were adjusted to match observed dengue incidence data.

To ensure consistency between the estimates of $\beta_{H}^{s},\delta_{i}$ and $sev_{_{i}}^{s},mil_{_{i}}^{s}$ we used an Expectation-Maximisation algorithm [11]: Each step of this algorithm includes the estimation of $\beta_{H}^{s},\delta_{i}$ based on an initial set of values for $sev_{_{i}}^{s},mil_{_{i}}^{s}$ and once the estimation is performed $sev_{_{i}}^{s},mil_{_{i}}^{s}$ are updated using estimation results. The process is repeated until convergence of $\beta_{H}^{s},\delta_{i},sev_{_{i}}^{s},mil_{_{i}}^{s}$ between two successive steps.

## Calibration of vector birth and biting rates

Vector birth rates, simplified into 12 month-specific values ($f_{V}^{m}$), were adjusted to match monthly vector density index derived from routine surveillance in Southern Vietnam ($dens^{m}$ with $\sum_{m=1}^{12} dens^{M}=12$) using the following formula (based on exponential growth/decay) .

$$f_{V}^{m}=\bar{f}_{V}+0.5\ln(dens^{m+1}/dens^{m-1})$$

In the equation above $\bar{f}_{V}$correspond to the annual average birth rate,

After adjusting the birth rates, monthly biting rates were adjusted by minimising the difference between observed monthly incidence and model output. Using data from January 1999 to December 2007, we therefore estimated a total of 108 month-specific biting rates ($\alpha_{V}^{ym}$). Dengue seasonality was then characterized by the average of the $\alpha_{V}^{ym}$ values over the 9 years of observation:

$$\bar{\alpha}_{V}^{m}=\sum_{y=1999}^{2007} \alpha_{V}^{ym}$$

## Comparison of model dynamics with observed annual dynamics

The dynamics of annual dengue incidence in Southern Vietnam were assessed using reported data presented in Figure 2. Two main aspects were considered: 1) periodicity defined through dominant cycles identified in Fourier spectral density [12] and Wavelet power spectrum [13] and 2) the range of variation using the coefficient of variation (standard deviation/average annual incidence). The Fourier and Wavelet power spectra corresponding to the observed annual incidence data are presented Figure S1.1


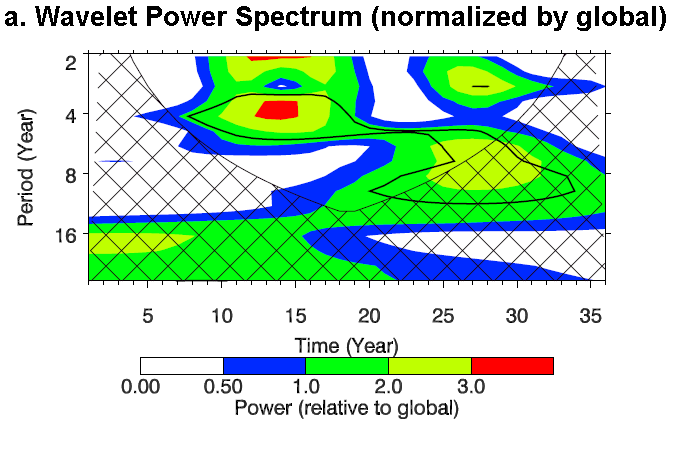


**Figure S1.1** **Spectral density in annual dengue incidence in Southern Vietnam. a**. Wavelet power spectrum using a morlet wavelet function. The power has been scaled by the global wavelet spectrum. The cross-hatched region is the cone of influence, where zero padding has reduced the variance. Black contour is the 10% significance level [13] **b**. Fourier Power Spectral density performed on detrented data with the spectrum function of the stats package of R [14].

Both the wavelet power spectrum and the Fourier power spectrum highlight the existence of 4-6 years cycles in the evolution of annual incidence. The coefficient of variation corresponding to the variation of annual incidence is 0.73

Model results do not exhibit fully stable dynamics. Therefore, for the two endpoints considered (dominant cycle and coefficient of variation) we calculated a range of variation by considering a reference period of 36 years (to match with available data) and used 136 years of model simulation.

# Latin hypercube design to explore serotype interaction scenarios

To explore the space of possible serotype interaction, we used Latin hypercube samples designed with the *optimumLHS* procedure of the R package lhs [15].

We limited the analysis to scenarios without intrinsic differences between serotypes (e.g. same increase in disease severity upon secondary infection, whatever the serotype) and associated with plausible parameter values. More specifically, we considered 3 groups of 50 scenarios:

1. Cross-protection only: For this group of scenarios we considered a duration of cross-protection ranging from 6 to 18 months ($\varepsilon_{H}$), an infectiousness in case of classical dengue fever $\left(\eta_{m}^{1},\eta_{m}^{2},\eta_{m}^{3},\eta_{m}^{4}\right)$ ranging from 1 to 5 times the infectiousness of asymptomatic infections, and infectiousness in case of severe disease i.e. dengue hemorrhagic fever $\left(\eta_{s}^{1},\eta_{s}^{2},\eta_{s}^{3},\eta_{s}^{4}\right)$ ranging from 1 to 3 times the infectiousness of classical dengue fever.
2. Cross-enhancement only: This group of scenarios is characterized by the absence of cross-protection ($\varepsilon_{H}=0$) but a range of infectiousness values identical to that of the first group. We also considered that, in case of secondary infection, the risk of being infected is increased by a factor of 1 to 5 ($\kappa_{x}^{s}$), the risk of developing mild disease is increased by a factor of 1 to 3 ($m_{x}^{s}$), and the increased risk of developing severe disease ($s_{x}^{s}$) is 1 to 3 times the increased risk of mild disease.
3. Cross-protection & cross-enhancement: For this group of scenarios and for all parameters ($\varepsilon_{H},\eta_{2}^{s},\eta_{3}^{s},\kappa_{x}^{s},m_{x}^{s},s_{x}^{s}$), we considered the same range of variations described above.

# Evolution of annual DHF/DSS incidence

Consistent with the approach adopted by Cuong et al. [16], we used a quasi-poisson regression for detecting the existence of a trend in the evolution of annual DHF/DSS incidence.

Table S1.3. Estimated annual change in dengue incidence based on quasi-poisson regression

There is a weakly significant positive trend (p-value =1.6%) when the entire period for which data are available is considered (Table S1.3). However, this trend is strongly linked to the incidence reported in the first years: the incidence in the first year is 70% lower than the next lowest value in the sample suggesting incomplete reporting. The trend is no longer significant at a 5% level if the first two years are removed from the sample. The absence of a trend is even more clearly seen if we consider the last 20 years for which the point estimate for the increase in the incidence rate is 0%.

Reference List

1. Hethcote HW (2000) The Mathematics of Infectious Diseases. SIAM Review 42: 499-633.
2. MacDonald G (1952) The analysis of equilibrium in malaria. Trop Dis Bull 49: 813-829.
3. Bartley LM, Donnelly CA, Garnett GP (2002) The seasonal pattern of dengue in endemic areas: mathematical models of mechanisms. Trans R Soc Trop Med Hyg 96: 387-397.
4. Huber K, Le Loan L, Hoang TH, Tien TK, Rodhain F, Failloux AB (2003) Aedes aegypti in south Vietnam: ecology, genetic structure, vectorial competence and resistance to insecticides. Southeast Asian J Trop Med Public Health 34: 81-86.
5. Tien NT, Luxemburger C, Toan NT, Pollissard-Gadroy L, Huong VT, Van Be P, Rang NN, Wartel TA, Lang J (2010) A prospective cohort study of dengue infection in schoolchildren in Long Xuyen, Viet Nam. Trans R Soc Trop Med Hyg 104: 592-600.
6. Vorndam V, Beltran M (2002) Enzyme-linked immunosorbent assay-format microneutralization test for dengue viruses. Am J Trop Med Hyg 66: 208-212.
7. Grenfell BT, Anderson RM (1985) The estimation of age-related rates of infection from case notifications and serological data. J Hyg (Lond) 95: 419-436.
8. Ferguson NM, Anderson RM, Garnett GP (1996) Mass vaccination to control chickenpox: the influence of zoster. Proc Natl Acad Sci U S A 93: 7231-7235.
9. Ferguson NM, Donnelly CA, Anderson RM (1999) Transmission dynamics and epidemiology of dengue: insights from age-stratified sero-prevalence surveys. Philos Trans R Soc Lond B Biol Sci 354: 757-768.
10. Rodriguez-Barraquer I, Cordeiro MT, Braga C, de Souza WV, Marques ET, Cummings DA (2011) From re-emergence to hyperendemicity: the natural history of the dengue epidemic in Brazil. PLoS Negl Trop Dis 5: e935.
11. Dempster A, Laird N, and Rubin D “Maximum likelihood from incomplete data via the EM algorithm,” J. Royal Statiscal Soc., Ser. R, vol.39, no.1, pp.1-38, 1977.
12. Brockwell, P. J. and Davis, R. A. (1991) Time Series: Theory and Methods. Second edition. Springer.
13. Torrence C, Compo GP (1998) A Practical Guide to Wavelet Analysis. Bulletin of the American Meteorological Society 79: 61-78.
14. Venables, W. N. and Ripley, B. D (2002) Modern Applied Statistics with S-PLUS, Fourth Edition. Springer.
15. Stocki R (2005) A method to improve design reliability using optimal Latin hypercube sampling. Computer Assisted Mechanics and Engineering Sciences 12: 87-105.
16. Cuong HQ, Hien NT, Duong TN, Phong TV, Cam NN, Farrar J, Nam VS, Thai KT, Horby P (2011) Quantifying the emergence of dengue in Hanoi, Vietnam: 1998-2009. PLoS Negl Trop Dis 5: e1322.
