# Supplementary material for: Transmission Dynamics of the Four Dengue Serotypes in Southern Vietnam and the Potential Impact of Vaccination
Source: PLoS One. 2012 Dec 10;7(12):e51244. doi: 10.1371/journal.pone.0051244 (PMC3519629; doi:10.1371/journal.pone.0051244)
Supplement: Text S2 — Supplementary results. (DOC) [file pone.0051244.s002.doc]

# Potential impact of vaccination on the transmission dynamics of dengue: a four serotype model

# Text S2 – Supplementary results

[S2.1 Summary tables 2](#__RefHeading___Toc332201943)

[S2.2 Models with cross protection only 6](#__RefHeading___Toc332201944)

[Model S2.2A 6](#__RefHeading___Toc332201945)

[Model S2.2B 12](#__RefHeading___Toc332201946)

[Model S2.2C 19](#__RefHeading___Toc332201947)

[S2.3 Models with cross-enhancement only 25](#__RefHeading___Toc332201948)

[Model S2.3A 25](#__RefHeading___Toc332201949)

[S2.4 Models with cross-protection and cross-enhancement 28](#__RefHeading___Toc332201950)

[Model S2.4A 28](#__RefHeading___Toc332201951)

[Model S2.4B 35](#__RefHeading___Toc332201952)

[Model S2.4C 41](#__RefHeading___Toc332201953)

[S2.5 Alternative scenarios of cross-interactions 47](#__RefHeading___Toc332201954)

[Cross-protection against symptomatic disease - S2.5A 47](#__RefHeading___Toc332201955)

[Temporary cross-enhancement - SC2.5B 50](#__RefHeading___Toc332201956)

[Full protection after two infections - SC2.5C 53](#__RefHeading___Toc332201957)

[No interaction between serotype - SC2.5D 56](#__RefHeading___Toc332201958)

[S2.6 Demographic changes 59](#__RefHeading___Toc332201959)

[High growth rate for host and vector population - S2.6A 59](#__RefHeading___Toc332201960)

[No growth for host and vector population - S2.6B 63](#__RefHeading___Toc332201961)

[High growth rate for the host population, no change for the vector population - S2.6C 67](#__RefHeading___Toc332201962)

[High growth rate for the vector population, no change for the host population - S2.6D 68](#__RefHeading___Toc332201963)

[Vector population -50%, 2 years - S2.6E 69](#__RefHeading___Toc332201964)

# Summary tables

**Table S2.1.1 Summary results for scenarios with cross-protection only**

**Table S2.1.2 Summary results for models with cross-enhancement only**

**Table S2.1.3 Summary results for models with cross-protection and cross-enhancement**

**Table S2.1.4 Summary results for alternative models of interactions between serotypes**

In the following sections, we present the detailed calibration and vaccination results for models providing the best match with observed periodicity for dengue in Southern Vietnam (model n°4, 30 and 49 among those only including cross-protection, model n°2, 16 and 23) for models combining cross-protection and cross-enhancement

We also present the detailed calibration results for the four alternative models for cross-interactions presented table S2.1.4 as well as for model n°1 with cross-enhancement only.

# Models with cross protection only

### Model S2.2A

#### Model Calibration

All result tables for this scenario are based on Model n°2 that provides the best fit of the data.

#### Vaccination results

### Model S2.2B

#### Model Calibration

All result tables for this scenario are based on Model n°2 that provides the best fit of the data.

#### Vaccination results

### Model S2.2C

#### Model Calibration

All result tables for this scenario are based on Model n°2 that provides the best fit of the data.

#### Vaccination results

# Models with cross-enhancement only

### Model S2.3A

# Models with cross-protection and cross-enhancement

### Model S2.4A

#### Model Calibration

All result tables for this scenario are based on Model n°2 that provides the best fit of the data.

#### Vaccination results

### Model S2.4B

#### Model Calibration

All result tables for this scenario are based on Model n°2 that provides the best fit of the data.

#### Vaccination results

### Model S2.4C

#### Model Calibration

All result tables for this scenario are based on Model n°2 that provides the best fit of the data.

#### Vaccination results

# Alternative scenarios of cross-interactions

### Cross-protection against symptomatic disease - S2.5A

### Temporary cross-enhancement - SC2.5B

### Full protection after two infections - SC2.5C

### No interaction between serotype - SC2.5D

# Demographic changes

### High growth rate for host and vector population - S2.6A

This scenario is based on model S2.4C (cross-protection & cross-enhancement) and considers an increase a high growth rate for both the host and vector population (3.4% i.e. twice the b aseline growth rate)

#### Vaccination results

### No growth for host and vector population - S2.6B

This scenario is based on model S2.4C (cross-protection & cross-enhancement) and considers a non-increasing host and vector population (growth rate 0%)

#### Vaccination results

### High growth rate for the host population, no change for the vector population - S2.6C

This scenario is based on model S2.4C (cross-protection & cross-enhancement) and considers a high growth rate for the host population (growth rate 3.4%) faster than the growth rate of the vector population (1.7%)

### High growth rate for the vector population, no change for the host population - S2.6D

This scenario is based on model S2.4C (cross-protection & cross-enhancement) and considers a high growth rate for the vector population (growth rate 3.4%) faster than the growth rate of the host population (1.7%)

### Vector population -50%, 2 years - S2.6E

This scenario is based on model S2.4A (cross-protection & cross-enhancement) and considers a sudden decrease of 50% of the vector population that ends after 2 years
